# Associations between private vaccine and antimicrobial consumption across Indian states, 2009–2017

**DOI:** 10.1111/nyas.14571

**Published:** 2021-02-05

**Authors:** Emily Schueller, Arindam Nandi, Jyoti Joshi, Ramanan Laxminarayan, Eili Y. Klein

**Affiliations:** ^1^ Center for Disease Dynamics Economics & Policy Silver Spring Maryland; ^2^ Center for Disease Dynamics Economics & Policy New Delhi India; ^3^ Amity Institute of Public Health Amity University Noida Uttar Pradesh India; ^4^ Princeton Environmental Institute Princeton University Princeton New Jersey; ^5^ Department of Emergency Medicine, Johns Hopkins School of Medicine, and Department of Epidemiology Johns Hopkins Bloomberg School of Epidemiology Baltimore Maryland

**Keywords:** India, vaccine, antibiotic use, antimicrobial use, antimicrobial resistance, AMR

## Abstract

Vaccines can reduce antibiotic use and, consequently, antimicrobial resistance by averting vaccine‐preventable and secondary infections. We estimated the associations between private vaccine and antibiotic consumption across Indian states during 2009–2017 using monthly and annual consumption data from IQVIA and employed fixed‐effects regression and the Arellano–Bond Generalized Method of Moments (GMM) model for panel data regression, which controlled for income and public sector vaccine use indicators obtained from other sources. In the annual data fixed‐effects model, a 1% increase in private vaccine consumption per 1000 under‐5 children was associated with a 0.22% increase in antibiotic consumption per 1000 people (*P* < 0.001). In the annual data GMM model, a 1% increase in private vaccine consumption per 1000 under‐5 children was associated with a 0.2% increase in private antibiotic consumption (*P* < 0.001). In the monthly data GMM model, private vaccine consumption was negatively associated with antibiotic consumption when 32, 34, 35, and 44–47 months had elapsed after vaccine consumption, with a positive association with lags of fewer than 18 months. These results indicate vaccine‐induced longer‐term reductions in antibiotic use in India, similar to findings of studies from other low‐ and middle‐income countries.

## Graphical abstract

The link between vaccines and reductions in antibiotic use is an emerging research area. In the United States and France, studies have found a reduction in antibiotic use associated with introduction of the seven‐valent pneumococcal conjugate vaccine. To improve understanding of the relationship between vaccines and antibiotic use, we examined the associations between consumption of vaccines and antibiotics from 2009 to 2017 in the private health sector in India, which has a lower.

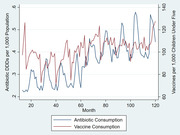

## Introduction

Although most high‐income countries (HICs) have greater access to antibiotics and greater consumption of antibiotics per capita than low‐ and middle‐income countries (LMICs),^1^ consumption in LMICs overall increased 56% between 2000 and 2015.^1^ India, which is projected to become the world's most populous country by 2024, had the greatest total consumption of antibiotics of any country in 2015, at 6.5 billion defined daily doses (DDDs), an increase of 103% from 2000.^1^ This is more than half the total of 10.3 billion DDDs consumed by all HICs.^1^, ^2^ Despite this high absolute consumption, India's antibiotic consumption rate of 13.6 DDDs per 1000 people per day is less than the global median of 19.5 and the rate of 25.7 in HICs.^1^

Rising consumption reflects improved access to antibiotics among patients in underserved communities within India, but overuse and misuse of antibiotics in communities with greater access remains a major challenge. A recent study of urban India estimated that more than 70% of children undergoing treatment for acute diarrhea may be inappropriately prescribed antibiotics.^3^ Inappropriate use can lead to antimicrobial resistance (AMR), leaving patients with fewer treatment options.^4^ Resistance to antibiotics, such as ciprofloxacin and ceftriaxone, has reached over 80% for some bacteria in India.^5^ AMR contributes to increased morbidity and mortality, especially among neonates;^6^, ^7^, ^8^ an estimated 58,000 neonatal sepsis deaths annually in India are attributable to antibiotic‐resistant bacteria.^9^

Routine childhood vaccination could substantially reduce the burden of vaccine‐preventable diseases and secondary infections, thereby reducing both appropriate and inappropriate use of antimicrobials and, in turn, lowering AMR.^10^, ^11^, ^12^, ^13^ Consequently, vaccination is an essential component in national and international strategies to reduce inappropriate antibiotic use and AMR.^14^, ^15^

The link between vaccines and reductions in antibiotic use is an emerging research area. In the United States and France, studies have found a reduction in antibiotic use associated with introduction of the seven‐valent pneumococcal conjugate vaccine (PCV).^16^ Similarly, in Finland, a trial of the 10‐valent pneumococcal vaccine found that antimicrobial use was 8% lower in the vaccinated group.^15^ A recent study found that a 10 percentage point increase in the influenza vaccination rate was associated with a 6.5% reduction in antibiotic use in the United States.^17^ Another study using household survey data from 77 countries found that the PCV and attenuated rotavirus vaccine were associated with 19.7% and 11.4% fewer antibiotic‐treated episodes of acute respiratory infection and diarrhea, respectively.^18^

Studies of the effect of vaccination on antibiotic use in LMICs are few, and results from HICs may not be generalizable to India, where the underlying burden of infectious diseases is higher, vaccination rates are lower, out‐of‐pocket expenditure on health is high, and access to antibiotics is often unregulated.^19^, ^20^, ^21^ For example, the effect of common vaccines, such as for measles or typhoid, which have been shown to provide additional nonspecific protection against diseases,^22^ cannot be determined in HICs where these vaccines are already widely available. To improve understanding of the relationship between vaccines and antibiotic use, we examined the associations between consumption of vaccines and antibiotics from 2009 to 2017 in the private health sector in India.

## Materials and methods

### Data

We used data on antibiotic and vaccine consumption from January 2009 to December 2017 obtained from IQVIA India.^23^ IQVIA uses sales surveys of private sector healthcare providers to develop state‐level estimates of the total volume of consumption of each antibiotic molecule (or combination of molecules) and vaccine. IQVIA's data collection methods are described in greater detail in the Supplemental Text (online only). Antibiotic consumption was reported for 13 states in India and Delhi, a government territory, for each month in 2009–2012 and for 21 states and Delhi for each month in 2013–2017. Data covered monthly and annual sales of 149 types of antibiotics.

Data on antibiotics delivered by public sector healthcare providers were not included in this analysis, as nationwide records were only available for gentamicin and co‐trimoxazole,^24^ which constituted a negligible proportion of the market overall.^25^ Nationally, 90% of all antibiotic sales in India are through private sector healthcare providers.^26^, ^27^ This proportion of market share has remained stable over time, as health care consumption has risen in both public and private sectors in India. While antibiotics can also be obtained through government‐run or affiliated clinics and hospitals, private providers account for 75% of outpatient and 62% of inpatient visits in India, mainly due to the poor quality and long wait times at public providers.^28^, ^29^, ^30^ As a result, the vast majority of antibiotic consumption is sourced from private providers.

To ensure comparability across formulations and products, antibiotic consumption data (i.e., grams) were converted to DDDs using the Anatomical Therapeutic Chemical Classification System (ATC/DDD, 2016) developed by the Collaborating Centre for Drug Statistics Methodology of the World Health Organization (WHOCC).^31^ For molecules not included in the ATC/DDD index, particularly fixed‐dose combinations (FDCs) that are unique to the Indian market, DDD values were estimated using an average of the constituent ingredients’ DDDs or as the average of DDD unit values by class, following previous methods used to compare IQVIA antibiotic consumption data across 76 countries.^1^ While other researchers have excluded FDCs from analysis of antibiotic consumption in India, following such an approach would have excluded a large amount of antibiotic consumption data from our analysis.^25^ Where the data indicated the name of the pack but not its strength, the number of grams in each drug was estimated through supplier descriptions on online marketplaces. Where the amount of active ingredient in a drug could not be directly ascertained, it was approximated through direct comparison with drugs of similar molecular composition and manufacture (Supplemental Text, online only).

Private vaccine consumption data were obtained from IQVIA based on surveys of vaccine sales in private pharmacies and facilities, and public vaccine consumption data were obtained from the Government of India Ministry of Health and Family Welfare's Health Management Information System (HMIS).^24^ We included only vaccinations intended to protect children against diseases that present with symptoms likely to be mistreated with antibiotics: measles, mumps, rubella, influenza, *Haemophilus influenzae* type b (Hib), rotavirus, diphtheria‐tetanus‐pertussis, *Streptococcus pneumoniae*, typhoid, and meningococcal meningitis. We considered the total number of doses of these vaccines, which were available annually and monthly in both the HMIS and IQVIA data.

Private sector vaccines account for an estimated 20% of all vaccines in India, and the remaining vaccines are delivered through the national routine childhood vaccination program known as the Universal Immunization Programme (UIP).^32^ The share of private sector vaccination varies across states; for example, for the oral polio vaccine, it ranges from 0.1% in West Bengal to 82% in Kerala.^32^ Because private sector vaccines are financed through out‐of‐pocket expenditures, their market shares are higher in wealthier states.^32^ The PCV and rotavirus vaccine were introduced gradually in UIP during 2015–2017 and were not widely available through the public healthcare system during the time period of our analysis.^33^, ^34^ The HMIS did not include data on these vaccines before their introduction, as the vaccines were only available in the private sector.

The primary source of state‐wise population data was the Indian census population projections.^35^ The census was conducted in 2011 before the creation of the state of Telangana in 2014. To account for the separation of one state (Andhra Pradesh) into two (Andhra Pradesh and Telangana), we assumed the proportion of population in each state remained constant before and after the separation. Monthly population estimates were constructed using constant linear growth rates year on year. Because the data preceded the creation of the union territory of Ladakh in 2018, we used population estimates for Jammu and Kashmir that did not account for this separation. Annual state‐wise income was obtained from the Reserve Bank of India's Handbook of Statistics on the Indian Economy and normalized to 2011 constant prices.^36^ For state‐wise analysis, annual income values were assigned to January and values for other months were interpolated with the assumption of constant linear growth in income from one year to the next.

Antibiotic consumption per 1000 people of all ages was calculated for each state or territory. Vaccine consumption rates were constructed per 1000 children under the age of 5 years in each state or territory.^33^ Although IQVIA only reported aggregate (all ages) antibiotic and vaccine sales data, analysis of the impact of childhood vaccination on overall antibiotic consumption is appropriate for several reasons. First, the 12 vaccines chosen in our analysis are for diseases that mainly affect under‐5 children and are often wrongly treated with antibiotics.^37^ Second, with the exception of tetanus vaccinations provided to pregnant women, vaccination of children over 5 years with booster doses and adult vaccination in India are negligible.^38^ While the full extent of adult vaccination in India is unknown and no national guidelines for adult immunization exist in India, evidence to date suggests very low uptake of adult vaccination.^39^ A 2013 study in Pune, India, found that only 8.3% of respondents were vaccinated against influenza during the H1N1 pandemic of 2009–2010, and a 2015 study of HPV awareness among students in a teaching hospital in south India found that only 6.8% of students surveyed had received the HPV vaccine.^40^, ^41^ Third, evidence from Canada, the United States, and the United Kingdom has shown that childhood vaccines may reduce the incidence of rotavirus and community‐acquired pneumonia in adult contacts of vaccinated children.^42^, ^43^, ^44^ Secondary protection provided by childhood vaccinations to other members of the household would reduce overall antibiotic use, and this would likely be greater in India than HICs because of lower overall adult vaccination coverage.

### Statistical analysis

We used fixed effects panel regression analysis to quantify the association between annual vaccination and antibiotic consumption rates. To capture the effect of socioeconomic status on capacity to access medication through the private sector, we included logged state‐wise income per capita. Standard errors were clustered at the state level. Fixed effects analysis was used only for annual data, as covariate data were not available in monthly increments.

To account for autocorrelation in antibiotic consumption, we also employed the Arellano–Bond Generalized Method of Moments (GMM) estimator, controlling for income. We evaluated the relationship between antibiotic consumption per capita and vaccination consumption per 1000 children under 5, allowing the time elapsed between vaccine and antibiotic consumption to vary, as vaccination provides long‐lasting protection against disease and existing evidence has suggested a reduction in antibiotic use in children aged 24–59 months due to vaccination.^18^ Analysis with annual data was limited by the number of observations, and we did not test models with more than a 2‐year difference between the metrics. Analysis with monthly data included up to 48 months between vaccine and antibiotic consumption. STATA version 16.1 was used for all statistical analyses, and results were considered significant at *P* < 0.05. Additional details of the model are presented in the Supplemental Text (online only).

## Results

**Figure 1:**
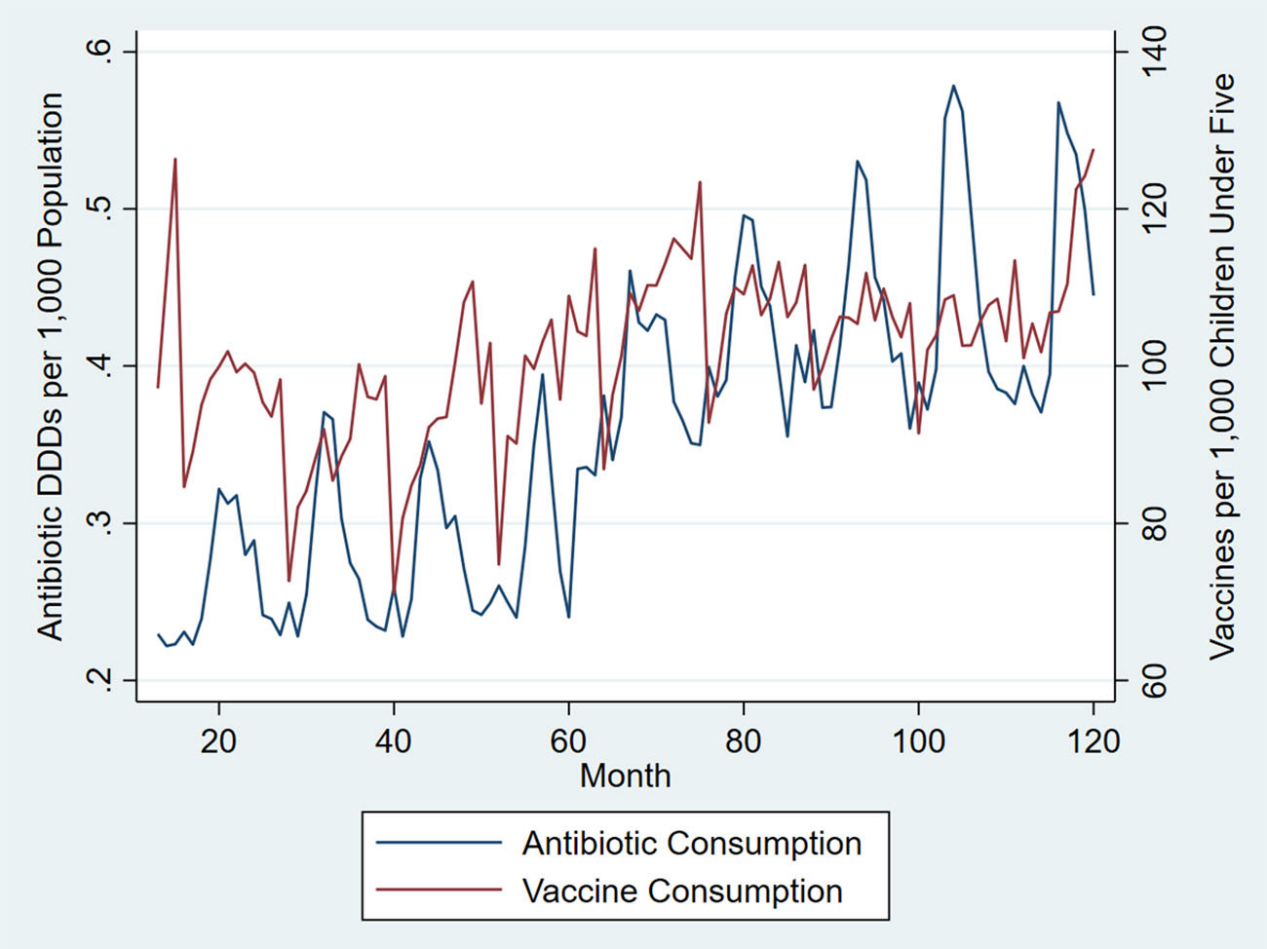
Monthly antibiotic and vaccine consumption in 13 Indian States and Delhi, 2009–2017. Sources: IQVIA 2018 and Government of India Health Management Information System. All rights reserved. DDDs were calculated using the Anatomical Therapeutic Chemical Classification System (ATC/DDD, 2016) developed by the Collaborating Centre for Drug Statistics Methodology of the World Health Organization (WHOCC). Indian states and Union territories include Andhra Pradesh, Assam, Bihar, Delhi, Gujarat, Karnataka, Kerala, Madhya Pradesh, Maharashtra, Odisha, Rajasthan, Tamil Nadu, Uttar Pradesh, and West Bengal. Other states were omitted due to lack of data before 2013.

**Figure 2:**
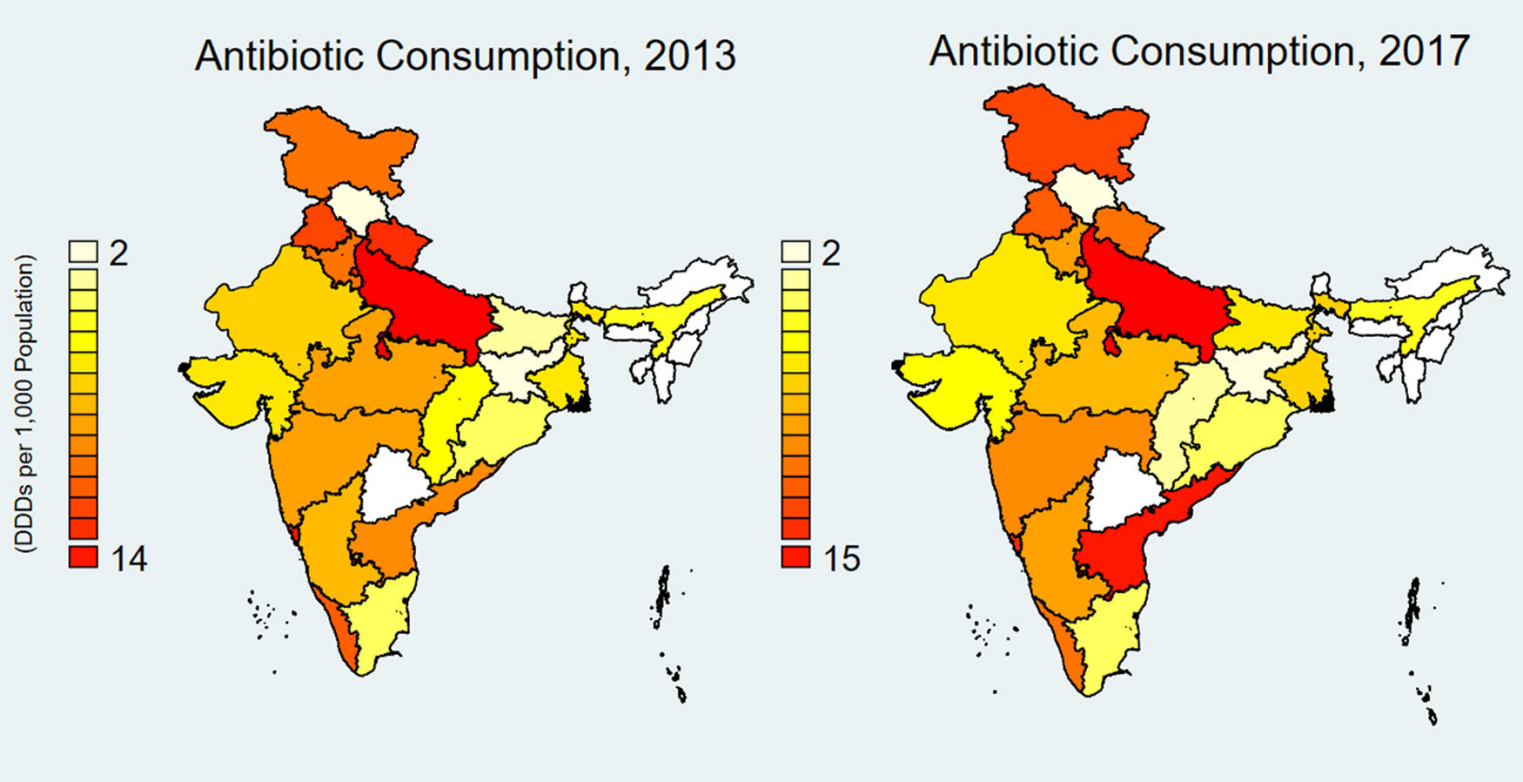
Private sector antibiotic consumption per 1000 population in 21 Indian States and Delhi, 2013 and 2017. Source: IQVIA 2018. All rights reserved. DDDs were calculated using the Anatomical Therapeutic Chemical Classification System (ATC/DDD, 2016) developed by the Collaborating Centre for Drug Statistics Methodology of the World Health Organization (WHOCC). Indian states and Union territories include Andhra Pradesh, Assam, Bihar, Chhattisgarh, Delhi, Goa, Gujarat, Haryana, Himachal Pradesh, Jammu and Kashmir, Jharkhand, Karnataka, Kerala, Madhya Pradesh, Maharashtra, Odisha, Punjab, Rajasthan, Tamil Nadu, Uttar Pradesh, Uttarakhand, and West Bengal. Other states were omitted due to lack of data.

**Figure 3:**
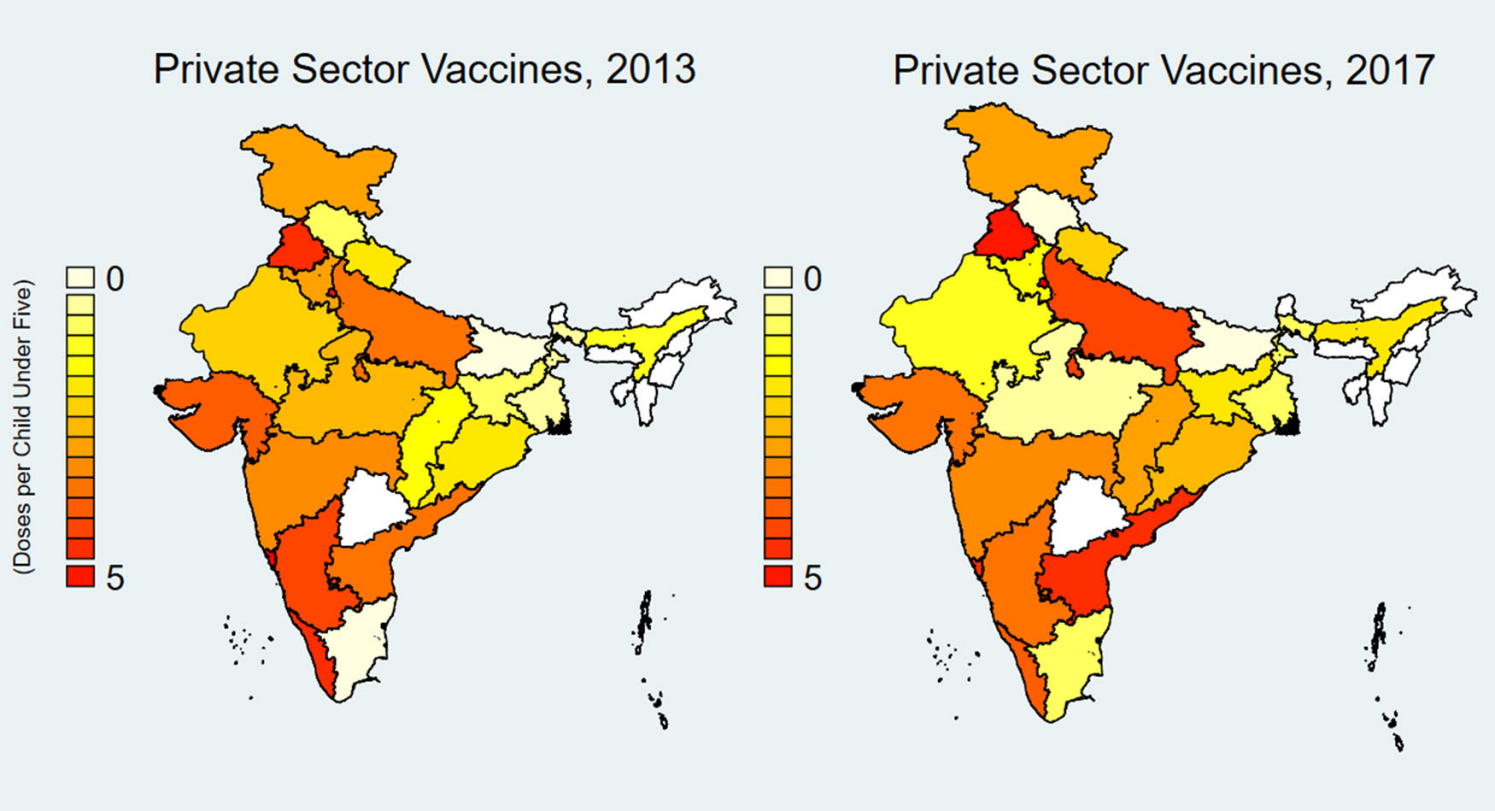
Private sector vaccine consumption per 1000 population in 21 Indian States and Delhi, 2013 and 2017. Source: IQVIA 2018. All rights reserved. Indian states and Union territories include Andhra Pradesh, Assam, Bihar, Chhattisgarh, Delhi, Goa, Gujarat, Haryana, Himachal Pradesh, Jammu and Kashmir, Jharkhand, Karnataka, Kerala, Madhya Pradesh, Maharashtra, Odisha, Punjab, Rajasthan, Tamil Nadu, Uttar Pradesh, Uttarakhand, and West Bengal. Other states were omitted due to lack of data.

Complete data were available for 15 states and Delhi for years 2009–2012 and for 21 states and Delhi for 2013–2017 (Table S1, online only). Overall, antibiotic consumption increased by 9.98% from 2009 to 2012 and by 13.4% from 2013 to 2017 but was highly seasonal (Fig. 1). Vaccine consumption increased after 2012 and was also seasonal, though it tended to peak earlier in the year than antibiotic use. Antibiotic and vaccine consumption varied substantially by state (Figs. 2 and 3). Both antibiotic and private vaccine consumption increased in the eastern region of India from 2013 to 2017.

### Analysis with monthly data

**Figure 4:**
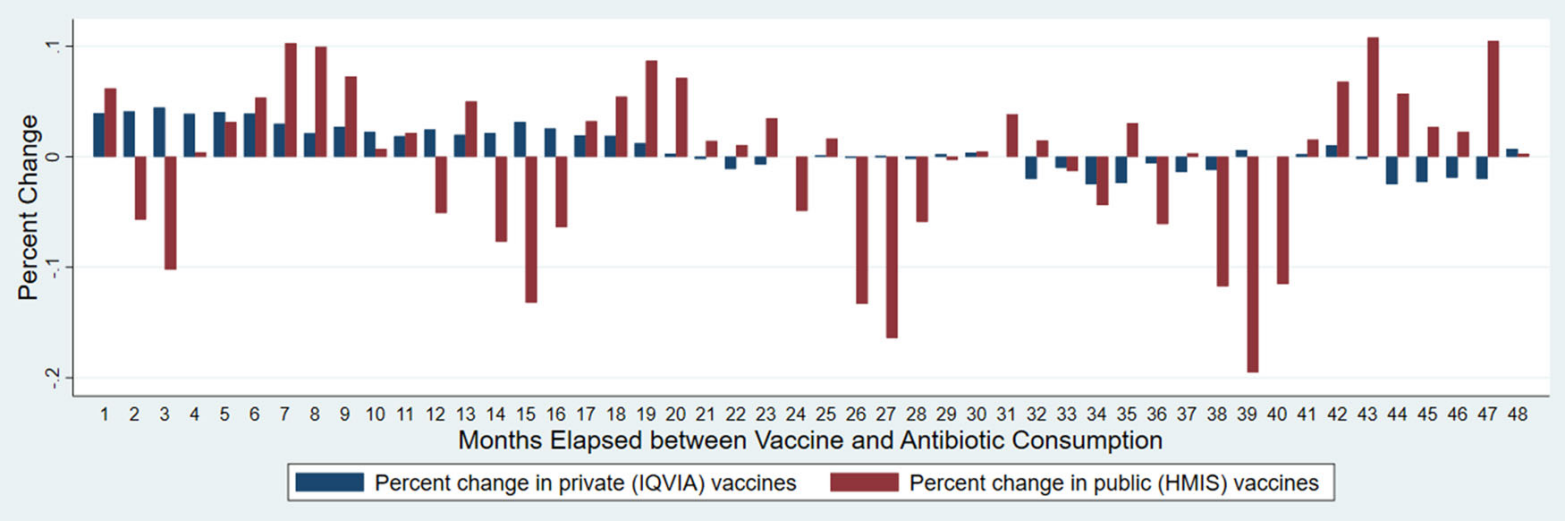
Arellano–Bond GMM estimation results: percent change in antibiotic consumption associated with a 1% increase in private sector vaccine consumption. Source: IQVIA 2018. All rights reserved. DDDs were calculated using the Anatomical Therapeutic Chemical Classification System (ATC/DDD, 2016) developed by the Collaborating Centre for Drug Statistics Methodology of the World Health Organization (WHOCC). For details on the model specification, see the Supplemental Text (online only).

In the Arellano–Bond GMM model, a 1% increase in private vaccine consumption was significantly associated with a 0.019–0.045% increase in antibiotic consumption with lags of fewer than 18 months (Table 1). At lags of 32, 34, 35, 38, 44, 45, 46, and 47 months, a 1% increase in private vaccine consumption was significantly associated with a 0.012–0.026% decrease in private antibiotic consumption (Fig. 4). Public vaccine consumption exhibited alternating positive and negative associations with antibiotic consumption, with positive associations at lags of 7, 8, 9, 19, 20, 43, 44, and 47 months and negative associations at lags of 14, 15, 16, 26, 27, 28, 39, and 20 months. (For all estimates and confidence intervals, see Table 1.) Positive associations ranged from a 0.0572% to 0.108% increase in antibiotic consumption per 1% increase in public vaccine consumption, and negative associations ranged from a 0.003% to 0.195% decrease. There were no significant associations with other lag values not mentioned here. Income was positively, significantly associated with antibiotic consumption for all evaluated lag values except for lags of 14 and 16 months.

**Table 1:** Arellano–Bond GMM estimation of percent change in private antibiotic consumption per 1000 people (monthly data)

Months elapsed between vaccine consumption and antibiotic consumption	Percent change in antibiotic consumption per 1% increase in private sector vaccine consumption per 1000 under‐5 children	*P* value	Percent change in antibiotic consumption per 1% increase in public sector vaccine consumption per 1000 under‐5 children	*P* value
1	0.040 [0.027:0.053]	<0.001***	0.062 [−0.001:0.125]	0.054
2	0.041 [0.028:0.054]	<0.001***	−0.058 [−0.136:0.021]	0.150
3	0.045 [0.031:0.059]	<0.001***	−0.102 [−0.205:0.002]	0.052
4	0.039 [0.027:0.051]	<0.001***	0.004 [−0.063:0.071]	0.913
5	0.040 [0.030:0.051]	<0.001***	0.031 [−0.026:0.088]	0.284
6	0.039 [0.027:0.051]	<0.001***	0.054 [−0.013:0.119]	0.111
7	0.030 [0.016:0.043]	<0.001***	0.103 [0.032:0.175]	0.005**
8	0.021 [0.008:0.034]	0.002**	0.100 [0.026:0.174]	0.008**
9	0.027 [0.016:0.038]	<0.001***	0.073 [0.006:0.140]	0.034*
10	0.022 [0.009:0.035]	0.001***	0.007 [−0.077:0.091]	0.878
11	0.019 [0.006:0.032]	0.005**	0.022 [−0.061:0.104]	0.619
12	0.025 [0.009:0.040]	0.002**	−0.051 [−0.116:0.014]	0.124
13	0.020 [0.006:0.034]	0.006**	0.050 [−0.014:0.114]	0.125
14	0.021 [0.010:0.033]	<0.001***	−0.077 [−0.117: −0.038]	<0.001***
15	0.031 [0.019:0.044]	<0.001***	−0.132 [−0.199: −0.065]	<0.001***
16	0.026 [0.014:0.037]	<0.001***	−0.065 [−0.120: −0.009]	0.022*
17	0.019 [0.008:0.031]	0.001***	0.032 [−0.029:0.094]	0.310
18	0.019 [0.003:0.035]	0.021*	0.054 [−0.010:0.118]	0.094
19	0.012 [−0.001:0.026]	0.072	0.087 [0.035:0.139]	0.001***
20	0.003 [−0.009:0.015]	0.650	0.071 [0.022:0.121]	0.005**
21	−0.002 [−0.014:0.009]	0.725	0.014 [−0.031:0.059]	0.545
22	−0.011 [−0.024:0.001]	0.073	0.011 [−0.046:0.067]	0.727
23	−0.007 [−0.024:0.009]	0.396	0.035 [−0.040:0.109]	0.365
24	−0.001 [−0.018:0.017]	0.942	−0.050 [−0.107:0.007]	0.089
25	0.001 [−0.013:0.015]	0.884	0.016 [−0.053:0.086]	0.657
26	−0.002 [−0.013:0.009]	0.757	−0.133 [−0.194: −0.073]	<0.001***
27	0.000 [−0.012:0.012]	0.979	−0.164 [−0.245: −0.084]	<0.001***
28	−0.003 [−0.014:0.008]	0.638	−0.060 [−0.113: −0.006]	0.029*
29	0.002 [−0.011:0.015]	0.742	−0.003 [−0.051:0.044]	0.894
30	0.004 [−0.012:0.020]	0.669	0.005 [−0.061:0.070]	0.897
31	−0.001 [−0.013:0.011]	0.890	0.039 [−0.017:0.094]	0.178
32	−0.020 [−0.032: −0.008]	0.001***	0.015 [−0.033:0.062]	0.561
33	−0.011 [−0.023:0.002]	0.094	−0.014 [−0.048:0.021]	0.455
34	−0.026 [−0.038: −0.014]	<0.001***	−0.045 [−0.092:0.002]	0.061
35	−0.024 [−0.034: −0.014]	<0.001***	0.030 [−0.028:0.089]	0.310
36	−0.006 [−0.018:0.005]	0.293	−0.061 [−0.124:0.002]	0.056
37	−0.015 [−0.031:0.001]	0.064	0.003 [−0.046:0.052]	0.913
38	−0.012 [−0.024: −0.001]	0.040*	−0.117 [−0.196: −0.038]	0.004**
39	0.006 [−0.007:0.020]	0.379	−0.195 [−0.311: −0.079]	0.001***
40	−0.000 [−0.011:0.010]	0.947	−0.115 [−0.191: −0.038]	0.003**
41	0.002 [−0.008:0.013]	0.681	0.015 [−0.038:0.069]	0.584
42	0.010 [−0.004:0.025]	0.157	0.068 [−0.000:0.136]	0.050*
43	−0.003 [−0.014:0.008]	0.642	0.108 [0.055:0.161]	<0.001***
44	−0.026 [−0.037: −0.015]	<0.001***	0.057 [0.000:0.114]	0.048*
45	−0.023 [−0.036: −0.011]	<0.001***	0.027 [−0.024:0.077]	0.300
46	−0.019 [−0.029: −0.009]	<0.001***	0.022 [−0.042:0.087]	0.507
47	−0.021 [−0.032: −0.009]	0.001***	0.105* [0.023:0.186]	0.011*
48	0.007 [−0.004:0.018]	0.203	0.003 [−0.103:0.109]	0.962

Coefficients represent the percent change in private antibiotic consumption per 1000 people due to a 1% increase in the private (or public) vaccine consumption per 1000 under‐5 children. Values in brackets indicate 95% confidence intervals. Significance levels of 10%, 5%, and 1% are denoted by ^*^, ^**^, and ^***^, respectively. The model included time effects for each month and income per capita, which had a positive and significant association with antibiotic consumption in every model except lags of 14 and 16 months. The model used the first differenced variable and two lags of the dependent variable.

### Analysis with annual data

In fixed‐effects analysis, a 1% increase in private vaccine consumption per 1000 children under 5 was associated with a 0.215% increase in DDDs per 1000 individuals in the same time period (Table 2). Similarly, in the Arellano–Bond model with annual data, we found that a 1% increase in private vaccine consumption was associated with a 0.204% increase in DDDs per 1000 individuals in the same time period (Table 3). Public vaccine consumption was not significantly associated with antibiotic consumption in any models with annual data. Logged income per capita was positively associated with antibiotic consumption in the fixed effects models but not in Arellano–Bond models.

**Table 2:** Fixed effects regression of private antibiotic consumption per 1000 people

	Log private antibiotic consumption (DDDs per 1000 people)	*P* value
Estimated percent change in antibiotic consumption per 1% increase in private sector vaccine consumption per 1000 under‐5 children	0.215 [0.136:0.293]	<0.001***
Estimated percent change in antibiotic consumption per 1% increase in public sector vaccine consumption per 1000 under‐5 children	0.161 [−0.099:0.421]	0.227
Estimated percent change in antibiotic consumption per 1% increase in income per capita (thousands INR)	0.471 [0.036:0.906]	0.034*
N	166	
*R* ^2^	0.798	

Coefficients represent the percent change in private antibiotic consumption per 1000 people due to a 1% increase in the private (or public) vaccine consumption per 1000 under‐5 children. For income per capita, the coefficient represents change in private antibiotic consumption due to INR 1000 increase. Significance levels of 5% and 0.1% are denoted by ^*^ and ^***^, respectively. DDDs were calculated using the Anatomical Therapeutic Chemical Classification System (ATC/DDD, 2016) developed by the Collaborating Centre for Drug Statistics Methodology of the World Health Organization (WHOCC). Source: IQVIA 2018, Government of India Health Management Information System. All rights reserved.

**Table 3:** Arellano–Bond GMM estimation of private antibiotic consumption per 1000 people (annual data)

	Percent change in antibiotic DDDs per 1000 people	*P* value
Estimated percent change in private antibiotic consumption (DDDs per 1000 people) per 1% change in current antibiotic consumption lagged 1 year	0.280 [0.119:0.442]	0.001***
Estimated percent change in antibiotic consumption per 1% increase in private sector vaccine consumption per 1000 under‐5 children	0.204 [0.150:0.258]	<0.001***
Estimated percent change in antibiotic consumption per 1% increase in public sector vaccine consumption per 1000 under‐5 children	0.175 [−0.009:0.360]	0.063
Estimated percent change in antibiotic consumption per 1% increase in income per capita (thousands INR)	0.051 [−0.249:0.351]	0.752
N	136	
Arellano–Bond test, order 1	−2.161	0.031*
Arellano–Bond test, order 2	−1.254	0.210

Coefficients represent the percent change in private antibiotic consumption per 1000 people due to a 1% increase in the private (or public) vaccine consumption per 1000 under‐5 children. For income per capita, the coefficient represents change in private antibiotic consumption due to INR 1000 increase. Significance levels of 5%, 1%, and 0.1% are denoted by ^*^, ^**^, and ^***^, respectively. DDDs were calculated using the Anatomical Therapeutic Chemical Classification System (ATC/DDD, 2016) developed by the Collaborating Centre for Drug Statistics Methodology of the World Health Organization (WHOCC). Source: IQVIA 2018, Government of India Health Management Information System. All rights reserved.

## Discussion

Access to antibiotics is limited in India, and children continue to die from treatable illnesses despite rapid growth in antibiotic consumption. An estimated 170,000 deaths of under‐5 children in India are caused by treatable infections every year.^45^ However, current patterns of antibiotic use indicate that when available, these drugs are often misused. A 2008 study of antibiotic prescribing practices in Uttar Pradesh found that more than 81% of patients in primary and secondary health care facilities were prescribed antibiotics, although facilities with more qualified staff and better infrastructure had lower prescribing rates.^46^ Antibiotics are widely available across India without physician prescription and often dispensed by pharmacists who lack formal training.^47^, ^48^, ^49^ A 2012 study in southern India found that half of observed pharmacies agreed to sell antibiotics without a prescription and over 60% of the pharmacists gave incorrect advice regarding antibiotic use.^48^ Although the Indian government banned the sale of 350 FDCs of antibiotics in 2016^50^ and requires prescriptions for the purchase of antibiotics through brick‐mortar (physical) or online pharmacies,^51^ the widescale purchase of banned antibiotic combinations has continued.^23^ Inappropriate use of antibiotics has been identified as a major driver of AMR.^52^ According to the Organization for Economic Co‐operation and Development, rates of resistance to certain bacteria exceed 90% in some LMICs.^53^ LMICs are particularly vulnerable to the effects of AMR due to limited access to water, sanitation, and hygiene, a high burden of infectious disease, and limited access to newer, more effective antibiotics.

Vaccines provide protection against primary and secondary infections from vaccine preventable diseases (VPDs), such as pneumonia, meningitis, and measles,^54^ and have been shown to decrease antibiotic use in several settings.^11^, ^15^, ^55^ Studies have found a negative association between vaccination rates and antibiotic use in HICs,^15^, ^56^ and emerging evidence suggests a similar association in LMICs. A 2020 study of antibiotic use in LMICs estimated that vaccination with 10‐ or 13‐valent PCV can avert 23.8 million episodes of antibiotic‐treated acute respiratory infection in children and vaccination with rotavirus vaccine can avert 13.6 million of antibiotic‐treated episodes of diarrhea annually.^18^

Most childhood vaccines provide long‐term or lifelong protection against infections. In an aggregate setting such as this study, the relationship between population‐level immunity from vaccines and the volume of antibiotic use may evolve over time. We modeled the prolonged effect by varying the number of months elapsed between vaccination and antibiotic consumption and found a negative association at higher lags. While most literature on the impact of vaccination on antibiotic use addresses this relationship over the course of a year or two, other studies have shown reductions in antibiotic use years later.^18^, ^57^, ^58^ Whether this relationship is causal is difficult to determine with certainty, as increased awareness of inappropriate antibiotic use, changes in clinical guidelines or practice, or consumer preferences in the health care market may also affect antibiotic consumption.

Increased levels of private sector antibiotic and vaccine consumption at low numbers of lags are likely driven by increased use of health care in the short term. This is supported by our finding that income exhibits a positive association with antibiotic consumption. Cyclical associations between public sector vaccination and antibiotic consumption may reflect the impact of regularly scheduled supplementary immunization activities at various points of the year or intermittent distribution of vaccines from the national to state governments. Additionally, efforts such as Mission Indradhanush—a large‐scale supplementary immunization program for underserved areas—shifted resources across states, likely resulting in temporal clustering of public sector vaccine delivery and changes in private antibiotic consumption. The relatively poor quality of public sector vaccination data and delays in reporting vaccine distribution figures to HMIS may also influence this relationship.

This analysis is novel in its use of Indian vaccine and antibiotic consumption data, and data availability posed limitations. Vaccine data were only available for 9 years, limiting the number of lagged values, which could be used in the annual Arellano–Bond GMM analysis. Data for antibiotic use in the public sector, which is estimated to represent 10% of overall antibiotic consumption,^25^ were not available for most states. The timing of the effect of childhood vaccination for long‐term VPDs on antibiotic consumption has not yet been established, so we varied the number of lags to account for this uncertainty. The nature of vaccine consumption in India presented another challenge, as Indian states spanned various stages of introducing rotavirus, PCV, and inactivated polio vaccine during 2009–2017.

Age‐disaggregated antibiotic sales were not available in our data. As a result, we could not discern how much of the long‐term reduction in antibiotic consumption can be attributed to the prevention of VPDs among children and how much may be due to secondary protection afforded to other adults in the household. Previous research has found a direct effect of vaccination on antibiotic use in children, but the indirect effects on antibiotic consumption in adults are not well established.^18^, ^57^ Additional research is needed to study potential spillover effects of child vaccination on illness and antibiotic consumption of caregivers. Finally, antibiotic sales are an imperfect proxy for actual antibiotic use by patients. Better quality data on antibiotic use are necessary to further study the relationship with vaccines.

The potential of vaccination to reduce unnecessary antibiotic consumption relies on large‐scale investments in childhood vaccination. A large majority of children from all communities must be vaccinated to achieve the necessary threshold for herd immunity and protect susceptible individuals against these diseases.^59^, ^60^ India has made progress toward this goal, but barriers remain. In 2014, Mission Indradhanush was launched with the goal of vaccinating more than 90% of all pregnant women and all children under 2 years of age in India against seven VPDs by 2020. Progress has been substantial but uneven: full immunization coverage—defined as children aged 12–23 months who received Bacillus Calmette–Guérin and measles vaccines and three doses each of polio and pentavalent vaccines—reached 89.1% in Punjab but only 47.1% in the northeastern state of Assam.^61^ Various factors, such as quality of public health facilities, level of education, and infrastructure, contribute to these disparities, with large differences in coverage between urban and rural areas.^62^, ^63^

Although the benefits of vaccination have been widely studied in terms of reductions in morbidity and mortality for patients, there is little research on its effect on antibiotic use in India. Our results indicate a negative relationship between monthly lagged vaccine consumption and monthly antibiotic consumption after a period of 32 months. However, this analysis was exploratory and limited by data availability. Further research, such as cohort‐based studies of vaccination and antibiotic use in children and comparisons of antibiotic use after the rollout of new vaccines, should examine long‐term secular trends and will further refine our understanding of vaccination as a potential mitigator of antibiotic consumption in India. Furthermore, changing patterns of health care‐seeking and the role of vaccines in specific antigens, coverage levels, and population groups must be considered as inappropriate antibiotic use continues. Vaccination must be used in tandem with other approaches, such as stewardship programs, to control infections and lower antibiotic use.

## Funding statement

This work was supported by the Value of Vaccination Research Network (VoVRN) through a grant from the Bill & Melinda Gates Foundation (OPP1158136). The content is solely the responsibility of the authors and does not necessarily reflect the views of the VoVRN or the foundation.

## Author contributions

A.N., J.J., R.L., and E.K. designed the study. E.S. and E.K. conducted the analysis and wrote the first version of the manuscript. All authors interpreted the findings and critically evaluated and edited the manuscript. All authors approved the final draft for publication.

## Competing interests

The authors declare no competing interests.

## Supporting information


**Table S1**. Data availability by Indian State or Union Territory.
