# Supplementary material for: Associations between private vaccine and antimicrobial consumption across Indian states, 2009–2017
Source: Ann N Y Acad Sci. 2021 Feb 5;1494(1):31–43. doi: 10.1111/nyas.14571 (PMC8248118; doi:10.1111/nyas.14571)
Supplement: Supplementary file 1 — Table S1. Data availability by Indian State or Union Territory. [file NYAS-1494-31-s001.docx]

**Supporting Information Appendix**

**for**

**The Association between Private Vaccine and Antimicrobial Consumption across Indian States, 2009-2017**

**Supplementary Methods**

**Data**

We used two datasets from IQVIA India: one from 2008 to 2012 and another from 2013 to 2017. The data include descriptions of each drug’s pack strength, active ingredient composition, and sales data regarding both money spent on the drugs (in crores, or tens of millions of Indian rupees) and thousands of packs sold. IQVIA samples distributors and sub-distributors that sell pharmaceutical products to dispensing doctors, pharmacists, and hospitals or other health facilities, which together account for an estimated 95 percent of the private pharmaceutical consumption in India. This stratified sample includes 1020 towns and all major metropolitan areas of India and approximately 27,500 individual distributors, from whom monthly sales data by product are collected. Number of packs sold and sales from each product are collected, along with details about the manufacturer, brand, type of molecule. Sales data are then anonymized, aggregated, and audited to ensure data obtained at different levels of the supply chain are counted correctly. While the data do not represent the quantity of antimicrobials and vaccines used by patients, IQVIA data present the most comprehensive pharmaceutical consumption data available across states in India.

WHO defined daily doses are provided in grams for most molecules; therefore, approximately 1,000 of the 13,500 unique combinations in the dataset needed to be searched individually to determine the number of grams in each active ingredient. MedPlus Mart, an online pharmacy and convenience store, carried a large majority of the pharmaceuticals in the dataset and was the primary source of pack strength data. Other pack strengths were found using India Mart, 1 MG, and MedIndia websites, among others. Many fixed-dose combinations (FDCs), particularly those combining penicillins and cephalosporins, were banned by the Indian government in 2016 and were discontinued in online marketplaces; in these cases, values of similar drug combinations were utilized.

India is home to many drug combinations that are not found in other countries and are therefore not included in the World Health Organization (WHO)’s guide to defined daily doses. In such cases, DDDs were determined by the active ingredients individually. Many of the drug combinations included a cephalosporin and a penicillin – in these cases, two measures were constructed for analysis, one using the DDD of each active ingredient. Results in Table 1 were reported for each FDC using the DDD value for penicillins. Results using the DDD value for cephalosporins did not differ substantially from those using the value for penicillins. Where the combination included two penicillins, amoxicillin or ampicillin was used to determine the value of DDDs. Where the combination included a penicillin and a fluoroquinolone, the penicillin was used to determine DDDs.

**Statistical Analysis**

We used the Arellano-Bond Generalized Method of Moments (GMM) estimator to evaluate the relationship between antibiotic consumption and vaccine consumption at various time intervals in the data. The Arellano-Bond GMM estimator is designed specifically for dynamic panel data that are stationary in their first differences, lack serial correlation in their errors, and demonstrate persistence in the dependent variable^1^. In this estimator, the dependent variable is differenced once to eliminate individual effects that may bias results, and lags of the dependent variable are used as instruments to account for endogeneity in the dependent variable, thereby eliminating omitted-variable bias due to endogeneity in the time series data. Additionally, the Arellano-Bond GMM estimator allows the intercept of each unit’s time series to vary using fixed effects while avoiding issues of endogeneity in traditional fixed-effects estimation ^2^. Thus, the Arellano-Bond GMM estimator is well-suited to our dataset, as the antibiotic consumption data demonstrate persistence in the dependent variable, stationarity in first differences, the absence of second order serial correlation, and autocorrelation and heteroskedasticity within individuals (in our case, Indian states and territories) but not between them^3^.

The primary limitation of the Arellano-Bond GMM estimator is potential weakness of its instruments, which may result in poor sample properties in terms of bias and consistency. This may be remedied through use of the system GMM estimator, which uses lagged first differences as instruments in a levels equation ^4,5^. However, system GMM estimation can lead to proliferation of instruments, with the number of instruments growing quadratically with T. Therefore, we used the first-difference Arellano-Bond GMM estimator.

The Arellano-Bond GMM estimator provides advantages to other commonly used methods for dynamic panel data analysis in this context. Panel vector autoregression (VAR) models suffer from bias when the number of observations (N) is large, but the time period (T) is small.^6,7^ The bias can remain even when T is as large as 30.^6,7^ Arellano-Bond GMM method can produce unbiased estimates at varying values of T as in our analysis.

We estimated panel VAR GMM models with fixed effects and found that the errors of the models exhibited substantial heteroskedasticity even after the inclusion of cluster-robust standard errors, while models using the Arellano-Bond GMM estimator did not. Furthermore, the Arellano-Bond GMM estimator allows the distribution of errors to vary as the estimator is asymptotically efficient, and it works well with unbalanced panel data compared to methods such as vector error correction models that focus on long-term cointegrated relationships.^8^

We consider an autoregressive dynamic panel model of order one of the type:

$y_{i,t}=\alpha y_{i,t-1}+{\beta X}_{i,t}+\gamma_{t}+\mu_{i}+\varepsilon_{i,t}$,

where the subscripts *i* and *t* denote Indian state or territory and year (in annual analysis) or month (in monthly analysis), $y_{i,t}$ is logged antibiotic consumption at time *t,* $y_{i,t-1}$ is one-period lagged logged antibiotic consumption, $X_{i,t}$ is a matrix of logged vaccine consumption (both public- and private-sector) and logged income per capita at time *t*, and $\gamma_{t}$ and $\mu_{i}$ are time effects and fixed effects, respectively. The error $\varepsilon_{i,t}$ is the idiosyncratic, random disturbance. The vector $X_{i,t}$ was varied to include different time values of public- and private-sector vaccine consumption and evaluate the effect of vaccine consumption on antibiotic consumption at varying time intervals.

In the case of our model, the data satisfy conditions necessary for Arellano-Bond GMM estimation, as the null hypothesis of serial correlation is rejected for order one and fails to be rejected for order two autocorrelation. Thus, we have

$E\left( \Delta y_{i\left( t-j \right)}\Delta\varepsilon_{it} \right)=0$ for all $j\geq2$

and the first-differenced lagged dependent variable is an adequate instrument. Furthermore, the Sargan test of overidentifying restrictions was used to determine the validity of the instruments. For all models, we failed to reject the null hypothesis that all instruments are valid.

**Supplementary Table 1: Data Availability by Indian State or Union Territory**

| **Indian State or Union Territory** | **Data Available 2008-2012** | **Data Available 2013-2017** |
| --- | --- | --- |
| Andhra Pradesh | Y | Y |
| Assam | Y | Y |
| Bihar | Y | Y |
| Chhattisgarh |  | Y |
| Delhi | Y | Y |
| Goa |  | Y |
| Gujarat | Y | Y |
| Haryana |  | Y |
| Himachal Pradesh |  | Y |
| Jammu and Kashmir |  | Y |
| Jharkhand |  | Y |
| Karnataka | Y | Y |
| Kerala | Y | Y |
| Madhya Pradesh | Y | Y |
| Maharashtra | Y | Y |
| Odisha | Y | Y |
| Punjab |  | Y |
| Rajasthan | Y | Y |
| Tamil Nadu | Y | Y |
| Uttar Pradesh | Y | Y |
| Uttarakhand |  | Y |
| West Bengal | Y | Y |

References

1 Arellano M, Bond S. Some Tests of Specification for Panel Data: Monte Carlo Evidence and an Application to Employment Equations. *The Review of Economic Studies* 1991; **58**: 277.

2 Canarella G, Miller SM. The determinants of growth in the U.S. information and communication technology (ICT) industry: A firm-level analysis. *Economic Modelling* 2018; **70**: 259–71.

3 Roodman D. How to do Xtabond2: An Introduction to Difference and System GMM in Stata. *The Stata Journal: Promoting communications on statistics and Stata* 2009; **9**: 86–136.

4 Arellano M, Bover O. Another look at the instrumental variable estimation of error-components models. *Journal of Econometrics* 1995; **68**: 29–51.

5 Blundell R, Bond S. Initial conditions and moment restrictions in dynamic panel data models. *Journal of Econometrics* 1998; **87**: 115–43.

6 Judson RA, Owen AL. Estimating dynamic panel data models: a guide for macroeconomists. *Economics Letters* 1999; **65**: 9–15.

7 Nickell S. Biases in Dynamic Models with Fixed Effects. *Econometrica* 1981; **49**: 1417–26.

8 Abrigo MRM, Love I. Estimation of Panel Vector Autoregression in Stata. *The Stata Journal* 2016; **16**: 778–804.
